# Evaluation and clinical practice of pathogens and antimicrobial resistance genes of BioFire FilmArray Pneumonia panel in lower respiratory tract infections

**DOI:** 10.1007/s15010-023-02144-2

**Published:** 2023-12-20

**Authors:** Jinru Gong, Jiasheng Yang, Lihong Liu, Xiaoxuan Chen, Guangyu Yang, Yaowei He, Ruilin Sun

**Affiliations:** 1 Department of Pulmonary and Critical Care Medicine, Guangdong Second Provincial General Hospital, Guangzhou, China; 2 The Second School of Clinical Medicine, Southern Medical University, Guangzhou, China

**Keywords:** BioFire FilmArray Pneumonia panel, Pneumonia, Conventional culture, Diagnostic efficacy, Clinical practice

## Abstract

**Background:**

Existing panels for lower respiratory tract infections (LRTIs) are slow and lack quantification of important pathogens and antimicrobial resistance, which are not solely responsible for their complex etiology and antibiotic resistance. BioFire FilmArray Pneumonia (PN) panels may provide rapid information on their etiology.

**Methods:**

The bronchoalveolar lavage fluid of 187 patients with LRTIs was simultaneously analyzed using a PN panel and cultivation, and the impact of the PN panel on clinical practice was assessed. The primary endpoint was to compare the consistency between the PN panel and conventional microbiology in terms of etiology and drug resistance, as well as to explore the clinical significance of the PN panel. The secondary endpoint was pathogen detection using the PN panel in patients with community-acquired pneumonia (CAP) or hospital-acquired pneumonia (HAP).

**Results:**

Fifty-seven patients with HAP and 130 with CAP were included. The most common pathogens of HAP were *Acinetobacter baumannii* and *Klebsiella pneumoniae*, with the most prevalent antimicrobial resistance (AMR) genes being CTX-M and KPC. For CAP, the most common pathogens were *Haemophilus influenzae* and *Staphylococcus aureus*, with the most frequent AMR genes being CTX-M and VIM. Compared with routine bacterial culture, the PN panel demonstrated an 85% combined positive percent agreement (PPA) and 92% negative percent agreement (NPA) for the qualitative identification of 13 bacterial targets. PN detection of bacteria with higher levels of semi-quantitative bacteria was associated with more positive bacterial cultures. Positive concordance between phenotypic resistance and the presence of corresponding AMR determinants was 85%, with 90% positive agreement between CTX-M-type extended-spectrum beta-lactamase gene type and phenotype and 100% agreement for mecA/C and MREJ. The clinical benefit of the PN panel increased by 25.97% compared with traditional cultural tests.

**Conclusion:**

The bacterial pathogens and AMR identified by the PN panel were in good agreement with conventional cultivation, and the clinical benefit of the PN panel increased by 25.97% compared with traditional detection. Therefore, the PN panel is recommended for patients with CAP or HAP who require prompt pathogen diagnosis and resistance identification.

**Supplementary Information:**

The online version contains supplementary material available at 10.1007/s15010-023-02144-2.

## Introduction

According to the World Health Organization report, lower respiratory tract infections (LRTIs) are the world’s deadliest infectious diseases and the fourth leading cause of death, with 2.6 million deaths reported in 2019 [1]. The pathogenic spectrum of respiratory infectious diseases is complex, and mixed infections are common with considerable heterogeneity [2, 3]. Delaying the timely and accurate treatment of pneumonia by even an hour leads to increased mortality [4]. However, traditional etiological methods, such as sputum culture with lengthy culture cycles, fail to detect coinfections and atypical pathogens. Currently, rapid molecular detection methods mostly target respiratory viruses, with limited focus on bacteria [5, 6]. Few assays are available for LRTI diagnostics [7–10]. Moreover, these combined panels lack semi-quantification of bacterial targets, which could enable the differential analysis of infection from colonization.

Antibiotic resistance exacerbates clinical complexity and increases mortality [11]. An estimated 4.95 million people died from bacterial antimicrobial resistance (AMR) in 2019, with 1.27 million deaths attributed to bacterial AMR [12]. Pathogen resistance in community-acquired pneumonia is also on the rise [13]. *Escherichia coli*, *Staphylococcus aureus*, *Klebsiella pneumoniae*, *Streptococcus pneumoniae*, *Acinetobacter baumannii*, and *Pseudomonas aeruginosa* are among the primary pathogens responsible for bacterial AMR-related deaths. Production of extended-spectrum beta-lactamase (ESBL) and carbapenem by multidrug-resistant bacteria is the drug-resistant mechanism that needs to be emphasized on, as β-lactam antibiotics constitute the most common drugs and account for 65% of the total antibiotic market [14]. Delayed detection of drug resistance can lead to pathogen dissemination in hospitals and an increase in treatment costs. However, traditional microbial diagnosis may take 3–4 days to 1 week for bacterial culture, strain isolation, and antimicrobial susceptibility testing (AST). Consequently, physicians are often compelled to prescribe antibiotics based on empirical guidelines and local epidemiological data. Accordingly, the detection of key AMR genes holds potential for the rapid identification of resistance information.

The BioFire FilmArray Pneumonia (PN) panel can concurrently detect 15 bacteria, 3 atypical pathogens, 9 viruses, and 7 drug-resistant genes through multiple PCR detections and has received approval from the US Food and Drug Administration (Supplementary Table S1). It incorporates sample preparation steps that limit manual operation time to < 5 min and can be run for approximately 1 h. This study aimed to assess the diagnostic value and potential clinical significance of the PN panel by comparing the consistency of etiology and drug resistance between the PN panel and traditional detection methods for acute LRTIs.

## Materials and methods

This prospective observational study was conducted at the Department of Pulmonary and Critical Care Medicine, Guangdong Second Provincial General Hospital, Guangzhou, China, between June 2021 and January 2023, and included patients diagnosed with acute LRTIs. Bronchoalveolar lavage fluid was collected using bronchoscopy and immediately underwent bedside surgery or was stored in a 4 °C refrigerator. This study received approval from the Ethics Committee of the Guangdong Second Provincial General Hospital (No. 2021-KY-167-02). All patients, or their guardians, provided informed consent. The inclusion criteria were (1) age older than 18 years old; (2) diagnosis of pneumonia according to the guidelines of the American Thoracic Society and the American Association for Infectious Diseases Society of America (ATS/IDSA) [2, 15]. Briefly, CAP occurs in the community, but HAP occurs in a patient who is not in the incubation period of a pathogenic infection but develops pneumonia within 48 h after admission [2]; (3) indications for bronchoalveolar lavage examination. The exclusion criteria were (1) fever and pneumonia caused by known non-infectious lung diseases, such as lung tumors, interstitial lung disease, pulmonary embolism, and other non-infectious pulmonary infiltrates; (2) contraindications to bronchial examination; (3) incomplete medical record information; and (4) lack of paired sputum culture.

### Routine bacterial culture

After collection, the samples were promptly sent to the central laboratory for qualitative cultivation according to the Technical guide WS/T 499-2017 of China [16]. Pathogenic bacteria that tested positive in culture were automatically identified using the Vitek 2 system. Following identification, antimicrobial susceptibility testing was performed using the broth dilution method. The drug sensitivity results of the positive cultures were interpreted as sensitive (S), intermediate (I), or resistant (R) according to the criteria [17].

### Metagenomic next-generation sequencing (mNGS)

mNGS primarily analyzes through the reversible terminator sequencing method. The process begins with nucleic acid extraction and concentration assessment of the biological sample’s DNA. Then, a DNA library is constructed, and a 50µL reaction system is established. This is followed by a series of PCR, after which the amplified products are sequenced using the NextSeq 550 platform. With the sequence data obtained, a database comparison is performed to ascertain the presence of any pathogens within the sample.

### BioFire FilmArray PN panel

The PN panel was operated in accordance with the guidelines provided by the manufacturer [18]. The detection reagent was fully enclosed and capable of completing DNA/RNA extraction, DNA/RNA purification, RNA transcription, nested PCR amplification, and real-time detection simultaneously. It also facilitated three repeated tests for each target and ensured comprehensive process quality control. Positive or negative results were identified by analyzing the melting curve of the pathogen, and an automated detection report was generated.

We utilized a semi-quantitative report of 10^4^–10^7^ genomic copies/mL to estimate the relative abundance of nucleic acids in these common bacteria. Absence of measurable amplification or a calculated value below 10^3.5^ copies/mL was deemed negative and reported as “undetected”. Viruses and atypical bacteria were qualitatively reported as “detected” or “undetected”. The presence of the AMR gene was also qualitatively reported as “detected” or “undetected”, provided that one or more relevant bacteria (i.e., potential carriers of the AMR gene) were detected in the sample. If no suitable bacteria were detected, the AMR gene result was reported as “N/A” (not applicable).

### Genotype-to-phenotype prediction of antibiotic resistance

When the same pathogen was detected using both the culture method and the PN panel simultaneously, we compared the consistency between genotype and phenotype resistance. If the PN panel detected the CTX-M gene and the bacterial culture results indicated resistance (including intermediate or resistant) to penicillins and first-, second-, or third-generation cephalosporins [19, 20], it was defined as consistent genotypic and phenotypic resistance of CTX-M. Otherwise, the results were considered inconsistent. If the PN panel identified one or more carbapenem resistance genes and the culture test suggested resistance to either meropenem or imipenem, the two results were considered consistent. If the PN panel detected the presence of mecA/C and MREJ resistance genes and the bacterial culture identified methicillin-resistant *S. aureus*, the drug resistance findings from both methods were considered consistent.

### Clinical benefits of the PN panel

After discharge, the microbiological etiology of each case and the clinical impact of PN panel were assessed by two senior physicians according to the clinical data. Based on the low sensitivity of culture to atypical pathogens and viruses, only bacterial PN panel are compared with cultural method. If the PN panel results for bacteria and culture were in positive or negative agreement, it indicated that the clinical detection efficacy of the two methods was comparable [21]. When the PN panel was in positive agreement with other PCR tests or mNGS but negative for culture, or even positive for culture but antimicrobial drugs were adjusted (such as initiation of targeted treatment, pathogen identification or treatment confirmation, treatment de-escalation) based on PN results, the clinical benefit of the PN panel was considered greater than that of the culture method. The PN panel was deemed to have no clinical benefit when clinical antibiotic adjustment was based on the culture method, regardless of the PN panel’s negative or positive results [22].

### Statistical analysis

Continuous variables are presented as median (interquartile range [IQR]) or mean (SD), while categorical variables are presented as numbers or numbers (percentages). Fisher’s exact test was used to compare categorical data. For continuous data, Student’s *t* test and non-parametric tests, such as the Mann–Whitney and Kruskal–Wallis tests, were used, as appropriate.

All statistical analyses were conducted using SPSS statistical software (version 23.0; IBM Corp.). The detection of targeted bacteria and AMR of the PN panel were compared with those of bacterial culture methods. Positive and negative percent agreements (PPA and NPA) were calculated as follows: PPA, the number of concordant positive results divided by the total number of positive results by culture-based methods; NPA, the number of concordant negative results divided by the total number of negative results by culture-based methods. The 95% confidence intervals were calculated using R software (version 4.0.3). Two-sided *P* values < 0.05 were considered statistically significant.

## Results

### Patient characteristics

**Fig. 1:**
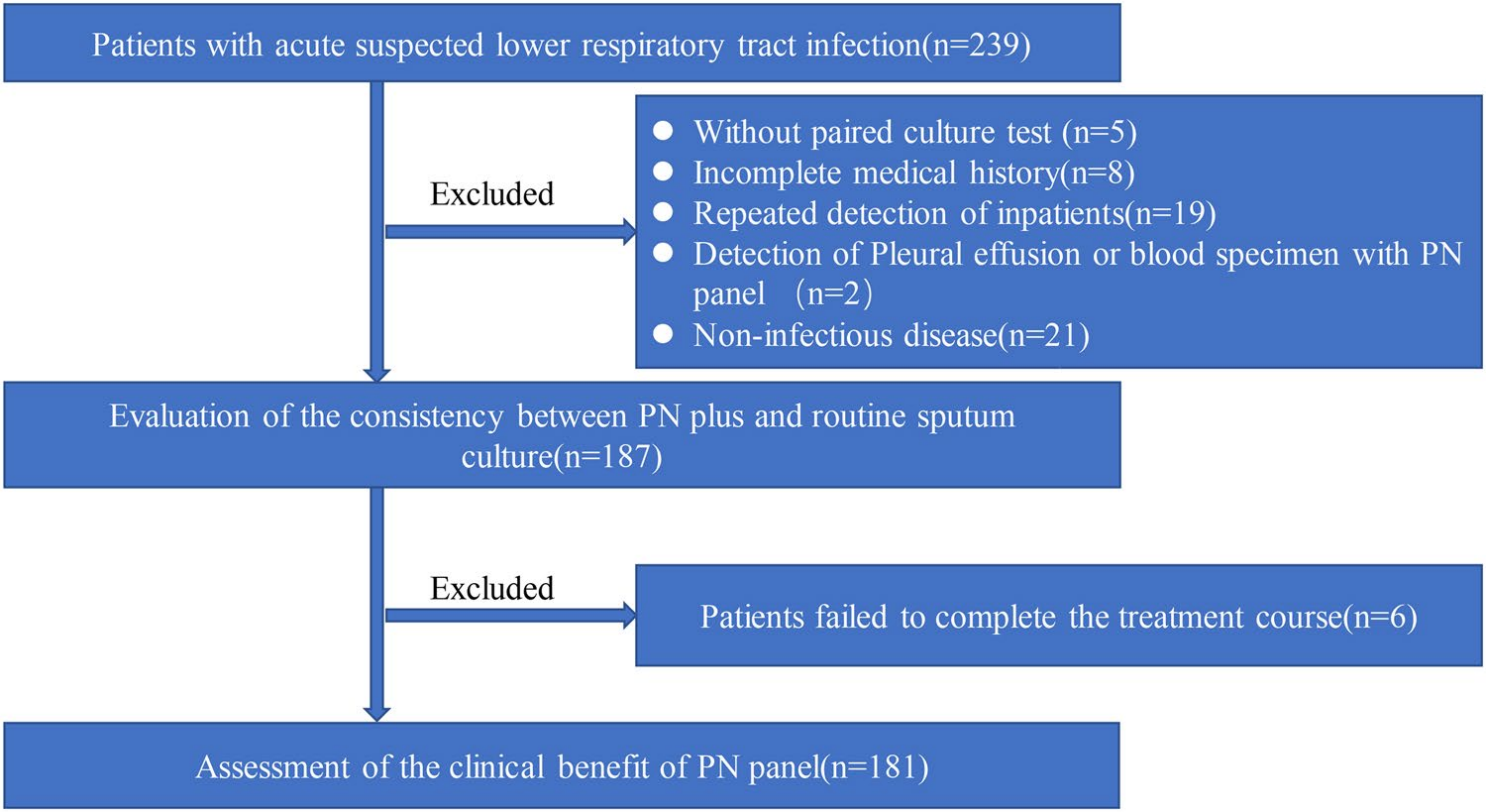
Flowchart of the study. A total of 181 patients completed the clinical efficacy evaluation using the PN panel test

This study initially included a total of 239 patients suspected of having acute LRTIs. However, 52 patients without a paired culture test, incomplete medical history, repeated sample detection, or with non-infectious diseases were excluded from the analysis. Consequently, 187 patients were included for the comparison of etiology and drug resistance genes, and 181 patients were evaluated for the clinical efficacy of the PN panel (Fig. 1).

Table 1 presents the demographic characteristics of the 132 male and 55 female participants included in the study. The average age was 64.00 ± 20.00 years, and 79.14% (148/187) of patients had underlying diseases, which included cardiovascular disease (19 cases), cerebrovascular disease (47 cases), Alzheimer’s disease (18 cases), diabetes (28 cases), hypertension (62 cases), chronic structural lung disease (49 cases), autoimmune diseases (9 cases), chronic kidney disease (10 cases), chronic liver disease (2 cases), malignant tumors (22 cases), indwelling endotracheal tubes (14 cases), and long-term bed rest (16 cases).

**Table 1:** Demographic characteristics and basic diseases

Subject	Cases (%)
Age (year, mean, SD)	64.00 ± 20.00
Male	132 (70.60%)
Comorbidity	n (%)
Diabetes	28 (14.98%)
Hypertension	62 (33.16%)
Alzheimer disease	18 (9.63%)
Structural lung diseases	49 (26.20%)
Cardiac disease	19 (10.16%)
Malignancy	22 (11.76%)
Cerebrovascular diseases	47 (25.13%)
Connective tissue disease	9 (4.81%)
Tolerance of endotracheal tube	14 (7.49%)
Long immobilization	16 (8.56%)

**Fig. 2:**
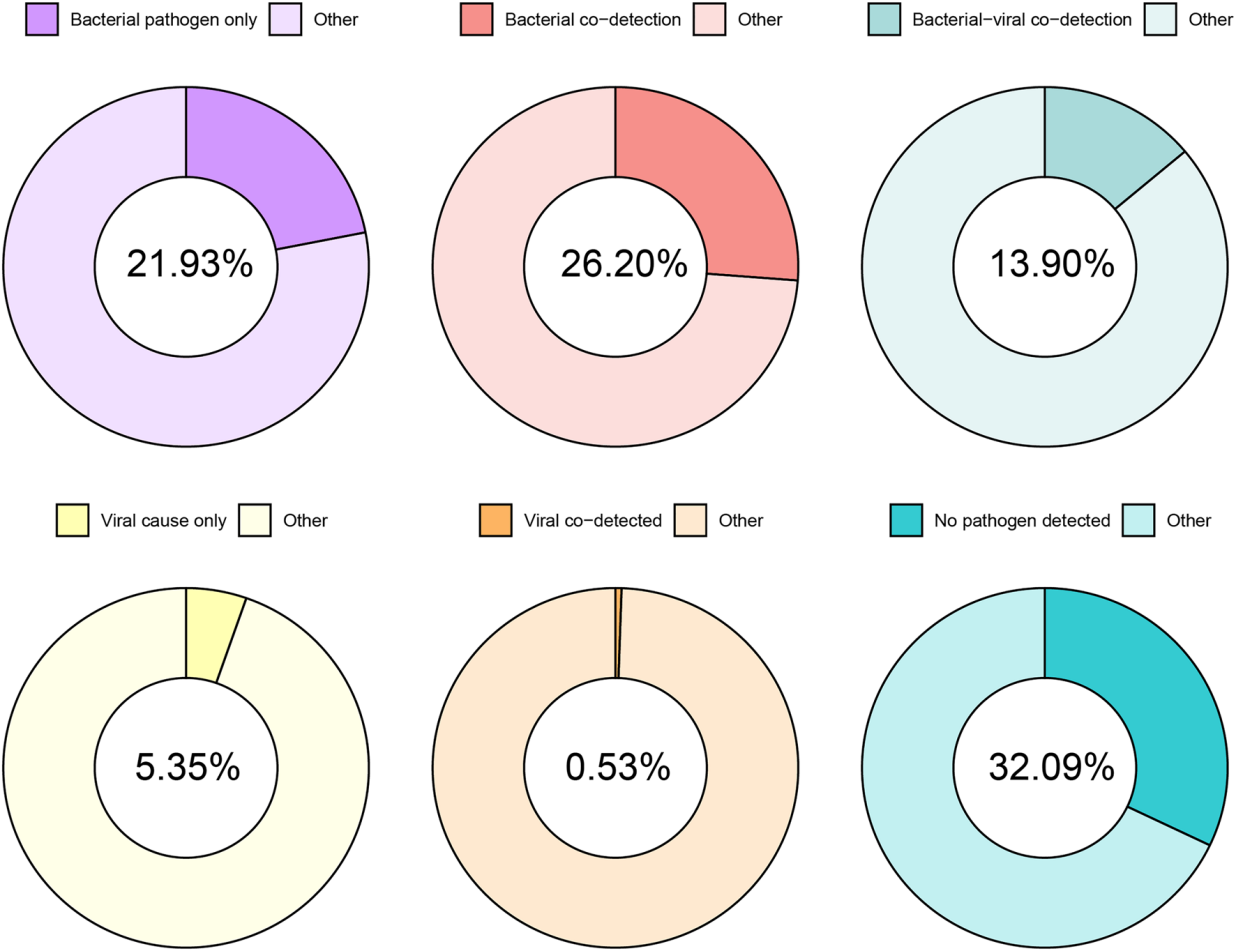
The ratio of pathogens detected in the PN panel bacteria were the predominant pathogens detected in 116 patients (62.03%). Bacterial and viral pathogens were detected simultaneously in 26 patients (13.9%)

The PN panel revealed that bacteria (including atypical bacteria) were the predominant pathogens, identified in 116 patients (62.03%). Concurrent detection of bacterial and viral pathogens was observed in 26 patients (13.9%). However, no pathogens were detected in 60 patients (32.09%; see Fig. 2).

### Detection of pathogens in patients with CAP and HAP using the PN panel

The PN panel demonstrated a significantly higher detection rate in the HAP group (51/57 [89.47%]) than in the CAP group (76/130 [58.46%]; *P* = 0.000). Additionally, the detection abundance in the HAP group was 2 (range 1–4), which was significantly higher than that in the CAP group (*P* = 0.000).

**Fig. 3:**
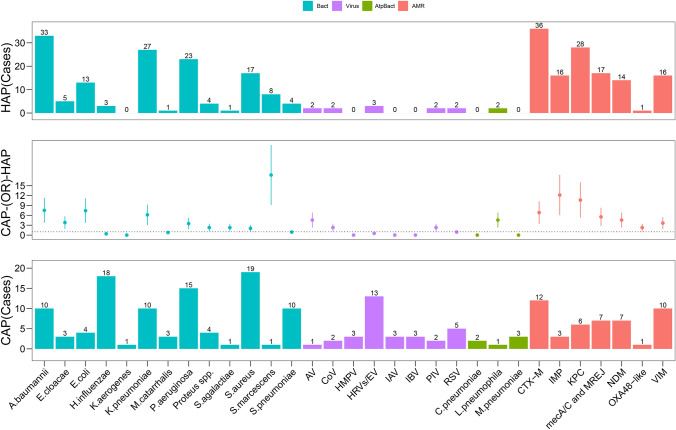
Differences in pathogens and AMR detected using the PN panel between patients with CAP and those with HAP. Blue bars represent bacteria, purple bars represent viruses, teal bars indicate atypical bacteria, and orange bars represent AMR genes

Among patients with CAP, *H. influenzae* (18/130 [13.85%]) and *S. aureus* (19/130 [14.62%]) were the most prevalent pathogens. In contrast, among patients with HAP, *A. baumannii* (33/57 [57.89%]) and *K. pneumoniae* (27/57 [47.37%]) were the most commonly identified pathogens. Figure 3 illustrates the discrepancies in pathogen detection between patients with CAP and those with HAP.

In the bronchoalveolar lavage fluid (BALF) samples from the HAP group, CTX-M and KPC were the most frequently detected AMR targets. In contrast, in the CAP group, CTX and VIM were the most commonly identified. Notably, the OXA48-like enzyme was the least detected in both groups.

### Comparison of detection results against bacteria and AMR between the PN panel and cultural methods

The typical positive bacterial rate of the PN panel (110/187 [58.82%]) was significantly higher than that of the cultural method (65/187 [34.76%]; *P* = 0.000). Table 2 displays the consistency of the bacterial detection results between the PN panel and culture methods. An assessment of the overall performance of the PN panel for the detection of bacterial targets revealed an 85% (51/60) PPA with routine culture results. The PPA for *H. influenzae*, *P. aeruginosa*, *S. marcescens*, and *S. aureus* was 100%, whereas that for *K. pneumoniae* was 73%. The NPAs ranged from 84 to 99%, with an overall NPA for bacterial detection of 92%.

**Table 2:** The concordance of bacteria between the Pneumonia panel and cultural methods

Tag	CM + PN +	CM + PN-	CM − PN +	CM − PN −	PPA (95%CI)	NPA (95%CI)
*A. baumannii* complex	19	4	24	140	0.83 (0.63–0.93)	0.85 (0.79–0.9)
*E. cloacae*	0	0	8	179	NA	0.96 (0.92–0.98)
*E. coli*	4	1	13	169	0.8 (0.38–0.96)	0.93 (0.88–0.96)
*H. influenzae*	1	0	20	166	1 (0.21–1)	0.89 (0.84–0.93)
*K. aerogenes*	0	0	1	186	NA	0.99 (0.97–1)
*K. pneumoniae*	11	4	26	146	0.73 (0.48–0.89)	0.85 (0.79–0.89)
*M. catarrhalis*	0	0	4	183	NA	0.98 (0.95–0.99)
*Proteus spp.*	0	0	4	183	NA	0.98 (0.95–0.99)
*P. aeruginosa*	9	0	29	149	1 (0.7–1)	0.84 (0.78–0.88)
*S. marcescens*	3	0	6	178	1 (0.44–1)	0.97 (0.93–0.98)
*S. aureus*	4	0	32	151	1 (0.51–1)	0.83 (0.76–0.87)
*S. agalactiae*	0	0	1	186	NA	0.99 (0.97–1)
*S. pneumoniae*	0	0	14	173	NA	0.93 (0.88–0.95)
Total	51	9	182	2189	0.85 (0.74–0.92)	0.92 (0.91–0.93)

Data are presented as n

*CM* cultural method; *PN* panel Pneumonia panel; *PPA* positive percent agreement; *NPA* negative percent agreement; *CI* confidence interval; *NA* the clinical features were not suitable for the control group

Table 3 presents the semi-quantitative results of the PN panel compared with the culture results. When the PN group exhibited an elevated semi-quantitative value of bacterial targets, the likelihood of consistently detecting the same positive bacteria using cultivation methods also increased. When the PN group detected bacterial targets of ≥ 10^7^ copies/mL, the proportion of positive bacterial culture was 45.83% (33/72), with *E. coli* accounting for 100%, followed by *K. pneumoniae* (76.9%), and the lowest consistent bacteria being *Proteus *spp. (0%) and *S. pneumoniae* (0%). When the PN group detected bacterial target values of 10^7^, 10^6^, 10^5^, and 10^4^ copies/mL, the proportions of positive bacterial cultures were 45.83% (33/72), 21.74% (10/46), 11.76% (8/68), and 0% (0/59), respectively (*P* = 0.000).

**Table 3:** The result of semiquantitative values of bacteria measured by the Pneumonia panel and the positive cultural method

Bacteria	Methods		Methods		Methods		Methods	
	PN (10^7^)	CM	PN (10^6^)	CM	PN (10^5^)	CM	PN (10^4^)	CM
*A. baumannii*	21	12	11	5	6	2	4	0
*Enterobacter cloacae complex*	0	0	0	0	4	0	3	0
*Escherichia coli*	3	3	6	0	3	1	5	0
*Haemophilus influenzae*	5	1	2	0	9	0	8	0
*Klebsiella aerogenes*	0	0	0	0	1	0	0	0
*Klebsiella pneumoniae* group	13	9	3	0	10	2	16	0
*Moraxella catarrhalis*	0	0	2	0	0	0	2	0
*Proteus* spp.	2	0	2	0	1	0	2	0
*Pseudomonas aeruginosa*	9	5	11	2	11	2	7	0
*Serratia marscens*	2	1	3	2	2	0	1	0
*Staphylococcus aureus*	11	2	5	1	13	1	10	0
*Streptococcus agalactiae*	0	0	0	0	1	0	0	0
*Streptococcus pneumoniae*	6	0	1	0	7	0	1	0
Total overall	72	33	46	10	68	8	59	0

Data are presented as n

*CM* cultural method; *PN* panel Pneumonia panel

### AMR genotype–phenotype associations

The PN panel and culture methods resulted in 51 cases of consistent bacterial identification, with *A. baumannii*, *K. pneumoniae*, and *P. aeruginosa* being the most frequently identified species. Among these, 46 cases exhibited potential carbapenemase and ESBL AMR in various bacteria, four cases involved *S. aureus*, and one case involved *H. influenzae*. Table 4 illustrates the qualitative comparison of bacterial targets between the BioFire FilmArray PN panel and culture methods. Out of the 30 strains demonstrating carbapenem resistance in the culture method, 24 strains exhibited carbapenem resistance genes in the PN panel. Overall, 90% (27/30) of bacterial strains detected using the PN panel indicated consistency between genotype and phenotype resistance for CTX-M genes. The PN panel accurately detected mecA/C and MREJ resistance genes when methicillin-resistant *S. aureus* was isolated using the culture methods, indicating that the PPA was 100%. Additionally, the NPA of mecA/C and MREJ was 100%.

**Table 4:** Consistency of genotype and phenotype resistance to antimicrobial resistance genes between the Pneumonia panel and the cultural methods

Tag	CM + PN +	CM + PN−	CM − PN +	CM − PN−	PPA (95%CI)	NPA (95%CI)
CTX-M^a^	27	3	2	14	0.9 (0.74–0.97)	0.88 (0.64–0.97)
Carbapenenase AMR^b^	24	6	12	4	0.8 (0.63–0.9)	0.25 (0.1–0.49)
mecA/C and MREJ^c^	1	0	0	3	1 (0.21–1)	1 (0.44–1)
Overall	52	9	14	21	0.85 (0.74–0.92)	0.6 (0.44–0.74)

Data are presented as n

*CM* cultural method; *PN* panel Pneumonia panel; *AMR* antimicrobial resistance; *PPA* positive percent agreement; *NPA* negative percent agreement; *CI* confdence interval

^a^resistance associated with CTX-M genes

^b^resistance associated with one or more carbapenenum resistance genes, including (KPC, NDM, Oxa48-like, VIM, IMP)

^c^resistance associated with mecA/C and MREJ genes

### Clinical practice of the PN panel

**Fig. 4:**
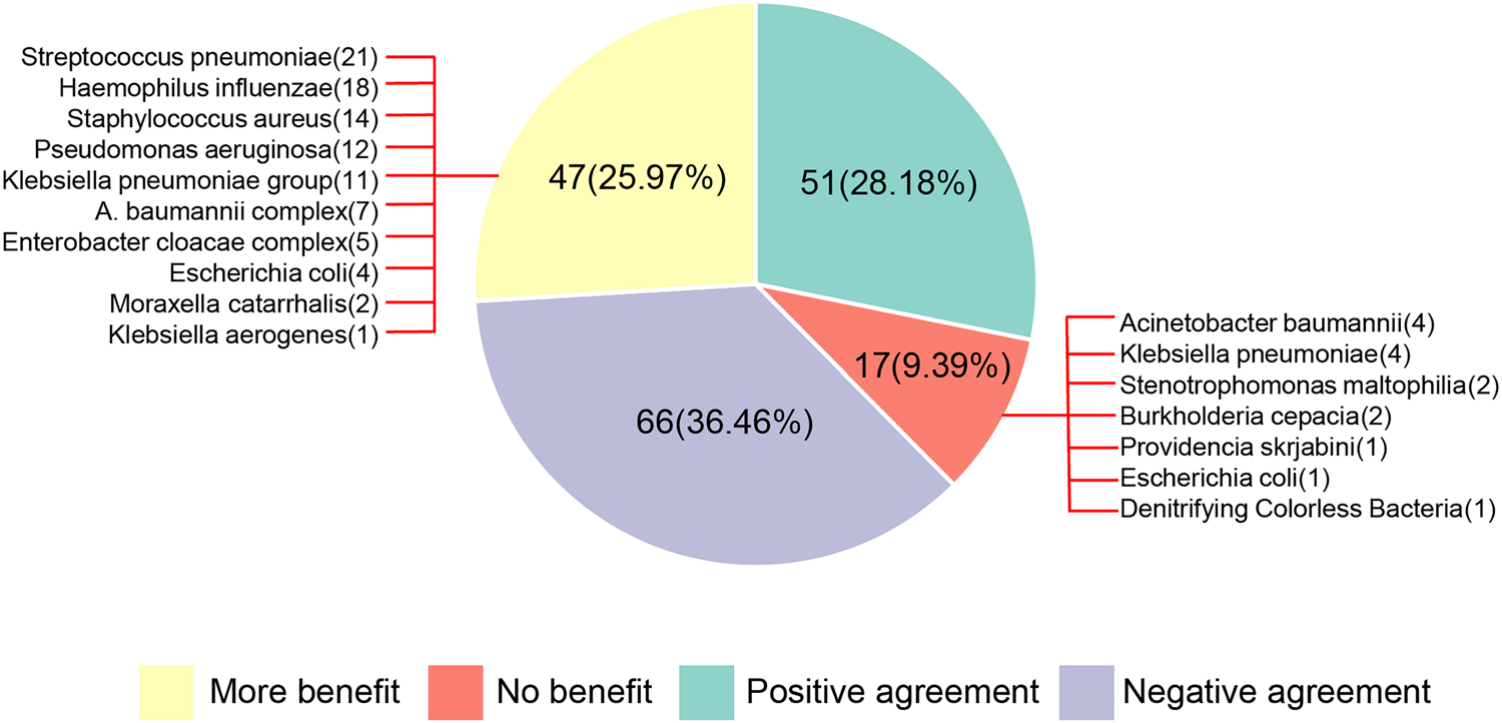
The clinical practice and missed pathogen of PN panel and culture method. The light green part represents positive agreement, the purple part represents negative agreement, the yellow part represents additional clinical benefit, and the red part represents no clinical advantages

Among the 181 patients who completed treatment, the PN panel consistently maintained negative results in 36.46% (66/181) of cases and consistently positive results in 28.18% (51/181) of cases compared with the culture methods. Compared with the culture methods, 25.97% (47/181) of patients experienced additional clinical benefits from the PN panel, while 9.39% (17/181) of patients did not experience any clinical benefit (Fig. 4). Among the pathogens that can demonstrate clinical benefits when using PN reports, the most frequently missed pathogens when using culture methods were *S. pneumoniae*, *H. influenzae*, *S. aureus*, *P. aeruginosa*, and *K. pneumoniae*. The pathogens missed by the PN panel were isolated from BALF samples using culture methods, including *A. baumannii*, *K. pneumoniae*, S. maltophila, *Burkholderia cepacia*, *Providencia skrjabini*, and *Achromobacter denitrificans.*

Among the 181 patients, there were 109 cases of bacterial detection (71 cases with AMR and 38 cases without AMR; Table 5). Hospitalization costs (*P* = 0.002) and antimicrobial drug use (*P* = 0.001) were higher in patients with drug resistance genes than in those without drug resistance genes. Compared with patients without AMR, those with AMR had a longer hospitalization time (14 days [interquartile range 10–19] vs 10 days [interquartile range 8–13]) and were more likely to develop complications (64.79% vs 21.05%). Adverse outcomes did not show significant differences between patients with and without AMR.

**Table 5:** Comparision of clinical outcome between patients with and without AMR genes

Outcomes	AMR (*n* = 71)	Non-AMR (*n* = 38)	*P*-value
Total hospitalization cost (RMB)	74,288.53 ± 6127.93	41,631.86 ± 7633.84	0.002
Antibiotic cost (RMB)	14,453.69 ± 1803.09	5971.02 ± 1109.07	0.001
Hospitalization duration (days)	14 (10.19)	10 (8.13)	0.010
Comorbidities (%)	46 (64.79%)	8 (21.05%)	0.002
Adverse outcome^a^ (%)	12 (16.90%)	3 (7.89%)	0.193

Data are presented as median (interquartile range [IQR]) or mean (SD) or n (percentage)

AMR,antimicrobial resistance

^a^death or exacerbation of illness transferred to the ICU

## Discussion

In this study, we designed a PN panel to evaluate the consistency between bacterial pathogens and AMR using routine culture methods. This is the first study to assess the significance of such a panel in clinical practice in China. Additionally, we analyzed the detection of the PN panel in cases of CAP and HAP. The bacterial panel and AMR both demonstrated a PPA of 85% compared with the conventional culture method. Apart from the advantages of point-of-care testing and rapid cultivation, the bacterial panel exhibited 25% more clinical benefits than the culture method. Also, early detection of AMR genes helps to adjust antibiotics in a timely manner, thereby avoiding complications and reducing the cost of antibiotics.

Bacteria were the most frequently detected pathogens in the PN panel, with *A. baumannii* and *K. pneumoniae* being the bacteria most strongly associated with HAP, in line with findings from previous studies [23, 24]. Unlike in a previous study, we found that *S. aureus* (14.62%) was more prevalent in patients with CAP than *S. pneumoniae* (7.69%), which might be related to the inclusion of nearly one-third of immunosuppressed patients in our study. As previously reported, *S. aureus*, *P. aeruginosa*, and *K. pneumoniae* are the most common bacterial infections in immunocompromised patients [25]. Hence, rapid bedside etiological testing is crucial as the etiology is linked to the patient’s underlying disease, disease severity, and even the site of onset. We observed that coinfection was more prevalent in patients with HAP than in those with CAP. Previous research has indicated that coinfection is significantly associated with disease severity and high mortality [26]. A study from Korea reported that 13.6% of patients with CAP had coinfection, while the proportion increased to 21.9% in patients with severe CAP [27]. The PN panel can simultaneously detect various bacteria, viruses, and even atypical pathogens, which may serve as an early warning indicator for the patient’s condition, whereas sputum bacterial cultures can only detect one pathogen at a time. In conclusion, the PN panel, as a multiplex detection reagent, may provide enhanced clinical practice guidance for physicians.

Consistent with the findings of a previous study [28], our study indicated that the PN panel detected more bacterial targets than the culture method, resulting in a 24.06% increase in patients reported as positive using the PN panel, with relatively high PPA (85%) and NPA (92%). Consequently, negative results may be employed for early antibiotic de-escalation, as the negative predictive value exceeded 90% in the PN panel. Similarly, prior research has reported superior performance of PN panels for bacterial detection, with PPAs ranging from 90.0 to 98.4% and NPAs ranging from 93.8 to 98.1% [21, 29–31]. While four *K. pneumoniae* strains were detected in the bacterial culture but not in the PN panel, resulting in a PPA of only 73%, *Stenotrophomonas maltophilia*, *B. cepacia*, and *Providencia skrjabini* were only detected using the culture method, whereas *S. pneumoniae*, *H. influenzae*, *S. aureus*, and *P. aeruginosa* were frequently missed.

Previous studies have demonstrated that quantitative PCR can distinguish symbiosis from pathogenicity by observing the charge [32], such as 10^3^ CFU/mL used for the protected specimen brush or 10^4^ CFU/mL of BAL used as an indicator to discontinue antibiotics against VAP [32]. The PN panel is semi-quantitative, with levels of 10^4^, 10^5^, 10^6^, and 10^7^ for bacterial targets, which is significant for guiding the initiation of antibiotic therapy in patients with HAP. In our study, an increasing number of semi-quantitative PN panels improved the likelihood of sputum cultures containing the same pathogen. The highest proportion of culture methods producing the same pathogen was observed when the PN panel detected bacterial targets of 10^7^ copies/mL or greater. Conversely, the corresponding pathogen was not detected in the sputum culture when the bacterial target was 10^4^ copies/mL, as detected using the PN panel. This suggests that it may be challenging to detect crucial organisms at extremely low concentrations, even though they are still associated with diseases. Further research is needed to explore whether the detection of unidentified, low-abundance, cultured microorganisms in the PN panel is of prognostic importance. Studies have also indicated that a high level of semi-quantitative signal intensity of positive microorganisms detected using multiple PCRs is closely related to positive bacterial cultures [29, 33], which may be useful for interpretation in the clinical applications of PN panels.

Molecular tests for genetic markers associated with antibiotic resistance, such as mecA, carbapenemases, and ESBLs, have been associated with positive outcomes, including reduced duration of optimal antibiotic therapy, shorter ICU stays, and decreased mortality rates [28, 34]. Our study demonstrated that hospitalization costs, antibiotic consumption, and the incidence of complications were higher in patients with drug-resistant genes than in those without drug-resistant genes. This emphasizes that early identification of drug resistance information and corresponding clinical interventions can help reduce economic costs and the occurrence of complications. Prior research has shown that the concordance rate for accessible resistance targets was 79% (14/18), consistent with phenotypic susceptibility testing [35], whereas in our study, the proportion of consistency in the phenotypic sensitivity test was 85% (52/61). Notably, mecA/C and MREJ of the PN panel exhibited extremely high predictive values for methicillin resistance, with 100% PPA and NPA in patients with positive *S. aureus* culture. Previous studies have indicated that the PPA for mecA/C and MREJ detection with PN panels was 100%, but NPA was < 90% [31]. However, further research is required to fully evaluate the PN panel, as our study included only four samples with positive *S. aureus* cultures.

We have previously described real-time PCR for the detection of NDM, KPC, VIM, IMP, and OXA-48, which are currently the most prevalent carbapenemase-producing genes [36]. In this study, the rate of phenotypic carbapenem resistance was relatively high, with 78.26% (36/46) of the specimens showing carbapenem resistance, the most common strains being *A. baumannii* and *K. pneumoniae*. Among the 30 carbapenem-resistant strains cultured, carbapenem-resistant genes were detected in 24 samples using the PN panels, while the remaining 6 were not detected. The six resistant strains were *P. aeruginosa* (three cases) and *A. baumannii* (three cases). This may be mediated by mechanisms other than carbapenem enzymes, such as the overexpression of efflux pumps or reduction of outer membrane pore proteins in *Pseudomonas* spp. [37]. Additionally, the overexpression of efflux pumps plays a significant role in the resistance of *A. baumannii* to tigecycline and imipenem [38]. Similarly, CTX-M testing demonstrated a positivity rate of 90%. However, 67% of these patients harbored concomitant carbapenemase genes. Considering that carbapenemase resistance often results in cephalosporin resistance [39, 40], the actual predictive efficacy of this measure may be diminished. These genetic tests facilitate the prompt addition of antibiotics and the implementation of appropriate isolation measures.

Our study has several limitations. First, we did not compare multiple specific etiological methods but rather culture results for bacteria with PN results. For example, bacterial culture is the primary method for the clinical diagnosis of *S. pneumoniae*; however, the detection rate of this method is relatively low and is influenced by various factors. Hence, urine antigen or other PCR tests should also be considered. Second, the methods of both analyses in this study were derived from the same alveolar lavage, but not the same specimen, which may have led to slight differences in the study results, although it is more in line with real-world research. Additionally, in this prospective study, we did not compare the clinical outcomes of the PN panel with those of standard methods. Our study revealed that patients in the HAP group had higher detection rates, a greater abundance of pathogens, and higher rates of resistance; however, the specific differentiation of clinical benefits was not achievable. Resistance genes influenced the clinical outcomes in our study, strongly supporting the necessity of detecting resistance genes in patients with LRTIs, though the cost of the panel will be higher than conventional culture. More prospective randomized studies are necessary to assess the impact of PN panels on the clinical outcomes of infected patients, including the types of pathogens and coinfections.

## Conclusions

In summary, the PN panel, functioning as a rapid molecular diagnostic technology, demonstrated superior performance in detecting and quantifying HAP pathogens compared to the CAP panel. The PN panel exhibited a higher microbial detection rate and abundance compared to conventional bacterial culture, with an overall agreement of 85%. The AMR indicators provided by the PN panel proved to be reliable predictors of bacterial resistance phenotypes, particularly in the case of *S. aureus*. Notably, the presence of these indicators was correlated with increased hospitalization costs and a higher incidence of complications.

### Supplementary Information

Below is the link to the electronic supplementary material.

Supplementary file1 (DOCX 16 KB)

## Data Availability

The datasets presented in this study are available upon reasonable request.

## Abbreviations

PN: Pneumonia
HAP: Hospital-acquired pneumonia
CAP: Community-acquired pneumonia
PPA: Positive percent agreement
NPA: Negative percent agreement
AMR: Antimicrobial resistance
AST: Antimicrobial susceptibility testing
IQR: Interquartile range
ATS/IDSA: American thoracic society and the American association for infectious diseases
BALF: Bronchoalveolar lavage fluid
ESBL: Extended-spectrum beta-lactamase
PCR: Polymerase chain reaction
Copies/mL: Copies per milliliter
