# Correction: Evaluation and clinical practice of pathogens and antimicrobial resistance genes of BioFire FilmArray Pneumonia panel in lower respiratory tract infections

**DOI:** 10.1007/s15010-024-02207-y

**Published:** 2024-02-15

**Authors:** Jinru Gong, Jiasheng Yang, Lihong Liu, Xiaoxuan Chen, Guangyu Yang, Yaowei He, Ruilin Sun

**Affiliations:** 1 Department of Pulmonary and Critical Care Medicine, Guangdong Second Provincial General Hospital, Guangzhou, China; 2 The Second School of Clinical Medicine, Southern Medical University, Guangzhou, China

**Correction: Infection** 10.1007/s15010-023-02144-2

In this article the affiliation details were incorrectly given as

1 Department of Pulmonary and Critical Care Medicine, Guangdong Second Provincial General Hospital, Guangzhou, China

2 Present Address: The Second School of Clinical Medicine, Southern Medical University, Guangzhou, China

but should have been

Jinru Gong#1, 2. Jiasheng Yang#1. Lihong Liu1, 2. Xiaoxuan Chen1. Guangyu Yang1, 2. Yaowei He1. Ruilin Sun*1,2

1 Department of Pulmonary and Critical Care Medicine, Guangdong Second Provincial General Hospital, Guangzhou, China

2 The Second School of Clinical Medicine, Southern Medical University, Guangzhou, China

*Corresponding author: Sun ruilin, sunruilin213@126.com

#Contributed equally.

