# Supplementary material for: Evaluation and clinical practice of pathogens and antimicrobial resistance genes of BioFire FilmArray Pneumonia panel in lower respiratory tract infections
Source: Infection. 2023 Dec 20;52(2):545–55. doi: 10.1007/s15010-023-02144-2 (PMC10955009; doi:10.1007/s15010-023-02144-2)
Supplement: Supplementary file 1 — Supplementary file1 (DOCX 16 KB) [file 15010_2023_2144_MOESM1_ESM.docx]

**Supplementary table S1. Targets of the Biofire pneumonia panel.**

| Category | Target |
| --- | --- |
| Bacteria (semiquantitative)^a^ | *Acinetobacter calcoaceticus-baumannii (A. baumannii )* |
|  | *Enterobacter cloacae (E. cloacae)* |
|  | *Escherichia coli (E. coli)* |
|  | *Haemophilus influenzae(H. influenzae)* |
|  | *Klebsiella aerogenes(K. aerogenes)* |
|  | *Klebsiella oxytoca (K. oxytoca)* |
|  | *Klebsiella pneumoniae group(K. pneumoniae)* |
|  | *Moraxella catarrhalis(M. catarrhalis)* |
|  | *Proteus spp.* |
|  | *Pseudomonas aeruginosa(P. aeruginosa)* |
|  | *Serratia marcescens(S. marcescens)* |
|  | *Staphylococcus aureus (S. aureus)* |
|  | *Streptococcus agalactiae (S. agalactiae)* |
|  | *Streptococcus pneumoniae (S. pneumoniae)* |
|  | *Streptococcus pyogenes(S. pyogenes)* |
| Atypical bacteria (qualitative) | *Legionella pneumophila(L. pneumophila)* |
|  | *Mycoplasma pneumoniae(M. pneumoniae)* |
|  | *Chlamydia pneumoniae(C. pneumoniae)* |
| Viruses (qualitative) | *Influenza A virus* (IAV) |
|  | *Influenza B virus*(IBV) |
|  | *Adenovirus*(AV) |
|  | *Coronavirus*(CoV) |
|  | *Parainfluenza virus*(PIV) |
|  | *Respiratory Syncytial virus*(RSV) |
|  | *Human Rhinovirus/Enterovirus*(HRVs/EV) |
|  | *Human Metapneumovirus*(HMPV) |
|  | Middle East respiratory syndrome (MERS-CoV) |
| Antimicrobial resistance genes |  |
| Extended-spectrum beta-lactamases | CTX-M^b^ |
| Carbapenemases | KPC^b^ |
|  | NDM^b^ |
|  | Oxa48-like^c^ |
|  | VIM^b^ |
|  | IMP^b^ |
| Methicillin resistance genes | mecA/mecC and MREJ^d^ |

a: Identified as 10^4^, 10^5^, 10^6^, or more than 10^7^ copies/ml.

b: Identified when *A. baumannii , E. cloacae ,E. coli, K. aerogenes, K. oxytoca, K. pneumoniae group, P. aeruginosa, S. marcescens or Proteus spp.* is also reported.

c: Identified when *E. cloacae complex, E. coli, K. aerogenes, K. oxytoca, K. pneumoniae group, S. marcescens or Proteus spp.* is also reported.

d: Identified when *S. aureus* is also reported.
